# A look back at a pilot of the citation typing ontology

**DOI:** 10.1186/s13321-023-00684-1

**Published:** 2023-02-03

**Authors:** Rajarshi Guha, Barbara Zdrazil, Nina Jeliazkova, Karina Martinez-Mayorga

**Affiliations:** 1 Vertex Pharmaceuticals, 50 Northern Ave, Boston, MA 02210 USA; 2 European Molecular Biology Laboratory, European Bioinformatics Institute (EMBL-EBI), Wellcome Genome Campus, Hinxton, Cambridgeshire, CB10 1SD UK; 3 Ideaconsult Ltd., Sofia, Bulgaria; 4 Institute of Chemistry, Campus Merida, National Autonomous University of Mexico, Merida-Tetiz Highway, Km. 4.5, Ucú, Yucatán Mexico; 5 Institute for Applied Mathematics and Systems, Merida Research Unit, National Autonomous University of Mexico, Sierra Papacal, Merida, Yucatan Mexico

Citations are a foundational component in scholarly writing, and while every citation has a reason for inclusion in a manuscript, the intent behind a particular citation needs to be inferred from the context. The Citation Typing Ontology (CiTO) was first described in [1, 2], motivated by the desire to allow authors to formally specify the intent of a given citation.

While it has seen relatively low usage in the scholarly publishing community since its introduction, there have been efforts to encourage its use, largely in a retrospective manner [3–6]. Given the utility of including machine readable annotations that would enhance the semantics of citations in journal articles, the Editors of the Journal of Cheminformatics initiated a pilot program to accept manuscripts containing CiTO annotations, as described in [7].

The pilot program ran from 2020 to 2022 and during this period published 15 articles, which annotated, on average, 75% of the bibliography with CiTO terms. A detailed analysis of the CiTO annotations included in the articles submitted to the Journal of Cheminformatics is presented in Wilighagen [8]. This editorial summarizes our experience with the pilot, in particular, some of the challenges we faced in working with CiTO annotations, and discusses our decision to discontinue the pilot.

While the ontology has existed for more than 10 years, it is not necessarily familiar to authors who would consider submitting to this journal. Thus we provided a landing page (https://www.biomedcentral.com/collections/cito) and guidelines (https://jcheminform.github.io/jcheminform-author-guidelines/cito) to authors wishing to include annotations in their submitted manuscripts. As noted there, we suggested a list of annotations for authors to consider, basing this list on the assumption that use of these annotations would be most informative. However, we did not restrict authors from using other annotations.

While it was encouraging that authors were responsive to the pilot, 15 articles corresponds to approximately 8% of all articles published in the period 2020–2022 and thus received relatively little attention over a two-year period. We recognize that submitting articles to this pilot involved a number of challenges.

First and foremost is that it required authors to familiarize themselves with the CiTO and be able to identify the most relevant terms for their citations. While we believe that it was not an overly onerous burden, it is still an additional task that must be considered when writing and submitting an article, requiring extra effort on the part of authors.

This leads to the second challenge. We required that submissions specify CiTO annotations as extra text appended to each bibliographic entry that authors intended to annotate. With the exception of CiteULike, we are not aware of any bibliography management tool that supports CiTO annotations. While a number of tools allow the user to the notes section of a bibliographic entry to specify the annotation, this is not native support. This meant authors needed to manually modify their final bibliography and insert CiTO annotations by hand. This is both tedious and an inefficient way to annotate citations. Nonetheless, it was interesting to note that on average authors annotated 75% of their bibliography (with 5 articles containing a 100% annotated bibliography).

The third challenge arose after authors submitted their articles. The journal publishing pipeline is not designed to support CiTO annotations and as a result required manual intervention at multiple points; editors needed to flag articles as part of a CiTO pilot, and processing of annotations also required manual intervention. This led to an inefficient article management and publishing pipeline, and led to errors in the final published form for some articles that subsequently needed to be corrected. As a consequence, the final publication of the articles including CiTO annotations was often delayed.

In summary, the inclusion of CiTO annotations was burdensome on authors and editors. More fundamentally, while the concept of formally annotating intent is commendable, it is unclear as to which is the real-world usage of such annotations. And thus the return on investment—for authors, editors and the journal—is unclear at this point in time.

Based on the relatively low response rate and the challenges described above, we have decided to end the pilot and will no longer officially support CiTO annotations. However, for authors who include CiTO annotations in their submissions, we will make an effort to ensure that they are considered in the publication process.

As recognized in [7], the use of CiTO is a chicken and egg problem, and we anticipate that in 2023, it still remains so. Nonetheless, we see a few actions that could help popularize the use of CiTO annotations and bring it into mainstream publishing.

First, there is a need for more education by journals and publishing organizations (academic and commercial) for authors. Second is CiTO support within bibliography management tools, as well as a recognition of CiTO annotations in standard bibliography formats. Finally, there is a need for a long term view on the part of publishers. While it is true that CiTO annotations do not have an immediate use case, the underlying premise is sound, and this is an effort where it seems that a “critical mass” of annotations must be achieved before any significant community-wide benefits can be seen. Essentially, this means that support for CiTO annotations requires an upfront investment in education and upgrading of publishing pipelines, without expectation of short term returns.

Hopefully publishers (and associated software developers) will recognize the scientific and social benefit that can accrue from a semantic representation of citations, and will be able to drive the uptake of CiTO in the future.

## Data Availability

The CiTO annotations for the articles in this pilot can be accessed from the individual articles.
